# Opto-Microfluidic Immunosensors: From Colorimetric to Plasmonic

**DOI:** 10.3390/mi7020029

**Published:** 2016-02-15

**Authors:** Jie-Long He, Da-Shin Wang, Shih-Kang Fan

**Affiliations:** Department of Mechanical Engineering, National Taiwan University, Taipei 10617, Taiwan; d93b47202@ntu.edu.tw (J.-L.H.); amydsw@gmail.com (D.-S.W.)

**Keywords:** optical detection, microfluidics, immunosensing

## Abstract

Optical detection has long been the most popular technique in immunosensing. Recent developments in the synthesis of luminescent probes and the fabrication of novel nanostructures enable more sensitive and efficient optical detection, which can be miniaturized and integrated with microfluidics to realize compact lab-on-a-chip immunosensors. These immunosensors are portable, economical and automated, but their sensitivity is not compromised. This review focuses on the incorporation and implementation of optical detection and microfluidics in immunosensors; it introduces the working principles of each optical detection technique and how it can be exploited in immunosensing. The recent progress in various opto-microfluidic immunosensor designs is described. Instead of being comprehensive to include all opto-microfluidic platforms, the report centers on the designs that are promising for point-of-care immunosensing diagnostics, in which ease of use, stability and cost-effective fabrication are emphasized.

## 1. Introduction

An immunosensor is an affinity-based sensor that exploits a specific interaction between an antigen and an antibody to detect and to quantify an analyte, which is typically an antigen that binds to an antibody immobilized on a sensor surface. In response to foreign molecules called antigens, the immune system produces antibodies to recognize antigens. Antigens are generally proteins, polysaccharides, small molecules (haptens) and even short peptides from an antigenic epitope, which might include parts of bacteria, viruses and other microorganisms. Monoclonal antibody (mAb) technology is an important scientific achievement; this process can produce many specific antibodies for immunoassays, which have been widely used for clinical diagnostics and basic biological research [1,2,3,4,5]. It is also a powerful tool for epidemiological surveillance of microorganisms, especially for influenza [6,7,8,9]. The binding strength between an antibody and its antigen can be represented with *K*_d_, a dissociation parameter, which typically lies in the nanomolar (nM) range [10]. A strong and specific interaction is the basis of a traditional immunoassay [11] and an emerging microfluidic immunosensor [12,13,14,15]. The production of high-quality antibodies or the design of alternative binding molecules and structures [16,17] hence plays a central role in deciding the specificity and sensitivity of an immunosensor. A subsequent immobilization on a sensor surface also affects the effective affinity. Because of the myriad design schemes and the chemical nature of this issue [18,19,20], an optimization of binding affinity is beyond the scope of this review, but one should note that a subsequent choice of transducer, which converts the bio-recognition event to a measurable signal, provides an additional rather than a decisive effect on the overall sensitivity of an immunosensor.

In contrast to a conventional immunoassay, which requires several repetitive steps with reagents, an immunosensor integrated with microfluidics has the advantage of automating and simplifying the steps to speed the measurement. Automation, speed, sensitivity and stability are the general indicators for the improvement of a sensor performance. Among detection methods of all types in immunosensing, which include electrochemical [21,22,23], optical [24,25], microgravimetric [26] and thermometric detection [27], optical detection is the mostly popular technique. The reason is its large ratio of signal to noise, non-destructive operation and rapid reading.

This review focuses on the introduction of several optical detection techniques and the microfluidic designs to facilitate the optical detection on a sensor. In Section 2 and Section 3, the working principles of labeled and label-free optical detection methods are reviewed; varied implementation with microfluidics is briefly introduced to help the reader understand how microfluidics can be integrated. Section 4 provides a detailed review of recent progress in microfluidic optical immunosensors. Despite numerous optical schemes that have been proposed for immunosensing in the past decade, few are commercialized and adopted in a clinical laboratory. An overview of recent advances in opto-microfluidic immunosensing have presented in Table 1. This review is hence not intended to be comprehensive to cover all recent progress in this field, but instead centers on the techniques widely adopted in medical laboratories and those emerging as potential alternatives in the future. The desired features for future medical application include instrumentation simplicity, less cost per assay, high sensitivity and specificity, less requisite sample volume and purity for assay, ease of multiplexingand, in particular, nonspecific background. Optical detections with signals produced by non-optical excitation such as enzyme-linked immunosorbent assay (ELISA) and electrochemiluminescence generally equip with simpler instrumentation while attaining high sensitivity; however, the multiplexing capability is compromised as not much signal modality can be separated for multiple analytes. Photoluminescence and surface plasmon resonance detection are particularly advantageous for multiplex screening, which require more sophisticated optical excitation and detection systems. To help the reader quickly get a flavor of these designs in advance, the major methods and their limits of detection are listed in Table 1.

**Table 1:** Comparison of various optical microfluidic immunosensing methods.

Detection Methods	Signal Generation	Label	Time Required	Microfluidic Device Types	LOD ^a^ Level	Ref.
Colormetric detection	Visible light illumination	Enzymatic catalyzed probes for ELISA	Minutes to hours	Microchannel	pg/mL	[28]
				Microchannel	pg/mL	[29]
				Strip-based	ng/mL	[30]
				Disc-based	ng/mL	[31]
Chemiluminescence	Chemical reaction	Enzymatic catalyzed probes for CL	Minutes	Opto-microfluidic	pg/mL	[32]
				Microchannel	pg/mL	[33]
				μPAD ^b^	ng/mL	[34]
				Disc-based	ng/mL	[35]
				EWOD ^c^	μg/mL	[36]
Electrochemiluminescence	Electrochemical reaction	ECL probes	Minutes	μPAD	ng/mL	[37]
				Microchannel	ng/mL	[38]
Photoluminescence	Optical excitation	Photoluminescent dyes	Real time	Microchannel	pg/mL	[39]
				Microchannel	pg/mL	[40]
				Microchannel	pg/mm^2^	[41]
				Microchannel	ng/mL	[42]
				EWOD	μg/mL	[43]
Surface plasmon resonance	Optical excitation with evanescent waves	Label-free	Real time	Microchannel	ng/mL	[44]
				EWOD	ng/mL	[45,46]

Notes: ^a^ LOD: Limit of detection. ^b^ μPADs: Microfluidic paper-based analytical devices. ^c^ EWOD: Electrowetting-on-dielectric.

## 2. Immunosensing with Optical Probes

**Figure 1:**
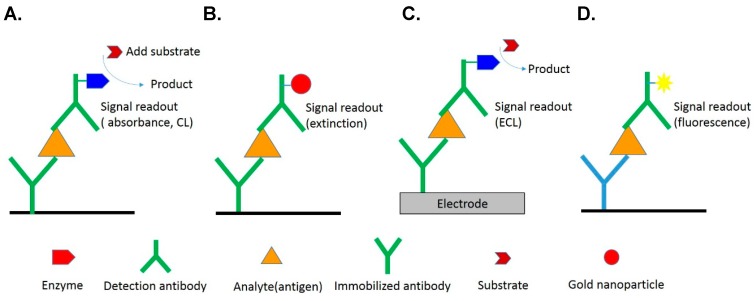
Comparison of immunosensing with optical probes. (**A**) Colorimetric and chemiluminescence (CL) induced by an enzyme; (**B**) Colorimetric changes by light absorption and scattering of a nanoparticle; (**C**) Electrochemiluminescence (ECL) from an electrochemically excited probe; (**D**) Photoluminescence from an optically excited probe.

Detection with an optical probe is the most common method in immunosensing [47]. The binding of an analyte with an immobilized antibody can be detected through an attached optical probe. An optical signal such as colorimetric detection, chemiluminescence (CL), electrochemiluminescence (ECL) and photoluminescence can be measured to detect analytes; this information is the physical basis for optical immunosensing. The optical signals are generated enzymatically or electrochemically or with optical excitation, depending on the choice of probe (See Figure 1).

### 2.1. Colorimetric Detection

The enzyme-linked immunosorbent assay (ELISA) that first appeared in the 1970s [48] used an enzyme rather than a radioactive label as the reporter. If the analyte is an antigen, an antibody is immobilized on the plate through adsorption. The detection is attained with another antibody conjugated (Figure 1A) with an enzyme that catalyzes the hydrolysis of a chromogenic substrate to render a colorimetric change measurable with a spectrophotometer, or simply visually. Common enzymes used in ELISA are horseradish peroxidase (HRP), alkaline phosphatase (AP) and β-D-galactosidase [28,49]. Washing and rinsing are incorporated after each incubation step to remove excess molecules and to eliminate non-specific binding. Apart from direct detection, ELISA schemes use a second antibody, which is a species-specific antibody that serves to recognize the first antibody. The second antibody is a detection antibody conjugated to the enzyme, as opposed to the first antibody in the direct method. Even though various solid-phase immunosensors are devised, ELISA performed on polystyrene microtiter plates is still the current standard in a medical laboratory; it is a sensitive technique with modest cost. The sensitivity arises from a signal amplification with enzyme-conjugates that allows the detection of minute concentrations of analytes, resulting in an improved detection limit to an ng level for biomarker detection [29,30,50].

The development of microfluidic ELISA has the advantages of saving the volume and duration of incubation of costly reagents. Lee *et al.* [31] developed a fully automated lab-on-a-disc (LOD) to perform microbead-based ELISA beginning with whole blood. An optical detector is equipped to quantify the colorimetric changes through absorbance. The use of microbeads increases the surface area and thus enhances the reaction efficiency, but microbeads are not easily retained in a suspension state; their transport hence cannot be accurately controlled with a continuous flow through a microfabricated channel. A disc platform provides an easy solution to this problem, in which the liquid transfer is controlled by the spin speed. With this LOD platform, the entire ELISA process from plasma blood separation, incubation of antibody, several steps of washing and final detection was attained automatically within 30 min using a drop of whole blood (150 µL), half the amount of a conventional ELISA. LOD immunosensing is hence an effective design for point-of-care (POC) laboratory settings [51].

### 2.2. Chemiluminescence (CL)

**Figure 2:**
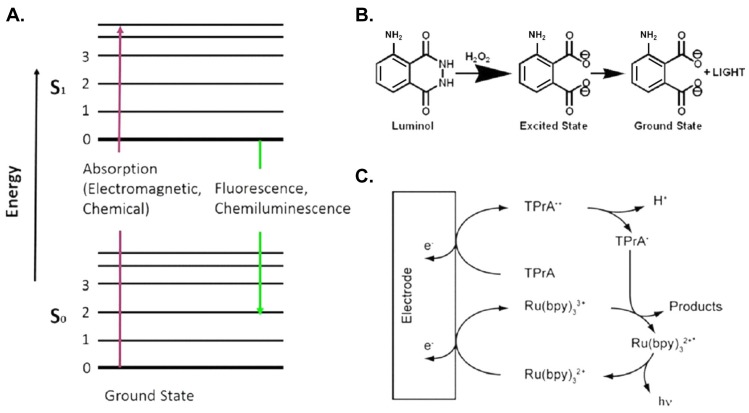
Mechanisms of luminescence. (**A**) Absorption of electromagnetic radiation, photoluminescence and chemiluminescence; (**B**) chemiluminescence; (**C**) electrochemiluminescence. Figure 2B is reproduced with permission from the Chemical Connection website [63].

Chemiluminescence (CL) signifies light emitted in a chemical reaction of a CL molecule [52,53], as opposed to optical excitation as in the case of photoluminescence, in which the emission is induced with absorbed electromagnetic radiation (Figure 2A). The electron of an organic molecule is excited to a higher energy state, with the energy provided by the oxidation reaction rather than absorption of radiation, and then processes following excitation hold both for chemiluminescence and fluorescence or phosphorescence, together known as photoluminescence. This chemical reaction is initiated with enzyme catalysis like ELISA. As CL requires no optical excitation, problems associated with photoluminescence such as scattering of excitation light or source instability are absent, as is also non-selective excitation such as background autophotoluminescence. The detection simply involves collecting sufficient photons from an inexpensive photomultiplier tube; no additional cost for excitation lamp and associated optics is required with a CL-based immunosensor. Another advantage of chemiluminescence is the large linear dynamic range. As it is an emission process, the optical signal is linearly proportional to the concentration; the linear dynamic range extends from a minimum detectable concentration up to six orders of magnitude [52]. For monoplex assay, CL detection with a photomultiplier tube is hence a superior choice for optical immunosensing in terms of sensitivity and cost [54,55,56].

A common chemiluminescent probe is luminol or acridinium ester [57]. The sensitivity might be increased through an accumulation of luminescent products and amplification of the enzymatic transduction signal [58,59]. The chemiluminescent reaction requires peroxide to attain an intermediate state; this intermediate decomposes with simultaneous light emission (Figure 2B). The quantum efficiency of this reaction is small, about 1%. Enzymatic catalysis is a way to accelerate the reaction and it thereby increases the rate of light emission. The enzymatic catalysis notably does not affect the quantum efficiency of the reaction, but simply increases the rate of the reaction cycle so that more light is accumulated within a given interval. Typical enzymes to catalyze the reaction are microperoxidase (MPO), myeloperoxidase and horseradish peroxidase (HRP). The CL immunosensing scheme is analogous to that of ELISA with the detection antibody conjugated to an enzyme. The chemiluminescent probe serves as the substrate to be catalyzed, as shown in Figure 1A.

Chemiluminescence is a promising detection method for immunosensing because of its simplicity, great sensitivity, small cost [34,60] and low-power demands [1,61]. The challenge for it to be implemented in a microfluidic device is the weak light from a small volume, which requires a sensitive optical detector such as a photomultiplier tube and a finely adjusted optical arrangement to acquire the signals. Pires *et al.* [32] reported a simple inexpensive opto-microfluidic device modified with gold nanoparticles that was equipped with a sensitive and stable organic photodetector. The gold nanoparticles enhanced the CL of the HRP-Luminol complex to attain a sensitivity 2.5 pg/mL, which is 200 times as sensitive as current CL immunosensors. Signal enhancement, more sensitive detectors and improved optical alignment and focusing are important factors to be developed for the incorporation of CL detection in a microfluidic device.

Yao *et al.* [33] integrated a magnetic microparticle technique and a CL immunoassay to develop an automated microfluidic system for rapid detection of insulin concentration. This system is expected to become a revolutionary platform in the clinical diagnosis of diabetes. Guo *et al.* [35] integrated a CD-type microfluidic platform and a CL immunoassay to detect a kind of endocrine disruptor, alkylphenolpolyethoxylates (APnEO). Zeng *et al.* [62] reported a portable prototype of a CL detector on an electrowetting-on-dielectric (EWOD) digital microfluidic platform, which has potential for development as being a highly sensitive, cheap and portable immunodetector.

### 2.3. Electrochemiluminescence (ECL)

Electrochemiluminescence (ECL) [64,65,66] pertains to chemiluminescence initiated in an electrochemical reaction. Among inorganic compounds known to be electrochemiluminescent, a widely exploited compound is a ruthenium complex, Ru(bpy)_3_^2+^. Ru(bpy)_3_^2+^ first proceeds through a redox reaction to produce Ru(bpy)_3_^3+^ and Ru(bpy)_3_^+^. In the presence of a co-reactant such as Tripropylamine (TPrA), Ru(bpy)_3_^3+^ converts to Ru(bpy)_3_^+^ and yields Ru(bpy)_3_^2+^ in an excited state that relaxes through photon emission as shown in Figure 2C. The reaction is efficient and can occur in an aqueous buffered solution with impurities and near 23 °C. These properties make it a popular ECL probe for immunosensing.

ECL has several advantages over conventional CL measurements. Because the reactants for ECL are produced by the applied potential, switching the applied potential can control the initiation and the course of a CL reaction. The “on and off” of CL light allows a user to discern the background stray light and include it into a correction. As the reactants are generated near the electrode and allowed to react immediately, the emission is concentrated near the electrode surface, which provides an accurate position for the subsequent optical alignment and focusing for maximum sensitivity. The intermediates of ruthenium complex can be converted back to Ru(bpy)_3_^2+^ after emitting light and are readily recycled at the electrode. The regenerated Ru(bpy)_3_^2+^ continuously participates the CL reaction in an excess of Tripropylamine, thus producing sufficient photons for detection. In addition, the recycling of reagent decreases the amount of reagents to be consumed while producing more photons per measurement cycle. ECL detection is more sensitive with more controllability than the conventional CL.

Because of the popularity of ECL immunosensing in a medical laboratory, a portable microfluidic ECL immunosensor is a promising device for POC examination. Wang *et al.* [37] introduced a battery-triggered microfluidic paper-based multiplex electrochemiluminescent immunosensor. Two ECL labels, Ru(bpy)_3_^2+^ and carbon nanodots, were used; four markers were detected using only two screen-printed working electrodes. Another modality of ECL detection [38], ECL resonance energy transfer (ECL-RET), in which the energy of ECL donor (Ru(bpy)_3_^2+^) transfers to that of an ECL acceptor (CdS nanorod), was adopted and proved to be highly sensitive for immunosensing. When a Ru(bpy)_3_^2+^/antibody was captured with an antigen on the CdS nanorod, the donor’s ECL emission decreased and a new emission signal emerged. Using a CdS nanorod as a spot site to capture the analyte and to initiate ECL-RET created a 64-site immunosensing array, making possible a multiplexed and sensitive ECL immunosensor.

### 2.4. Photoluminescence

The light emitted in photoluminescence is similar to chemiluminescence, which involves the transfer of electronic energy from the first excited state S_1_ to the ground state S_0_ with a photon emission in the visible light region (see Figure 2A). The difference lies in that photoluminescence is generated from an optical excitation. Through the nature of optical excitation, several problems must be solved to attain great sensitivity, which includes the separation of excitation and detection, the filtering of scattered light, background photoluminescence from optical components, cuvettes and reagents, and photoluminescent quenching [67]. As the quantum efficiency of photoluminescent emission is generally sufficiently large to produce enough photons for detection, it can serve directly as a probe without addition of an enzyme as in the case of CL [42,68]; this effect makes it suitable for imaging use and multiplex sensing [69,70,71]. There is also a large selection of photoluminescent probes, such as membrane-bound, calcium-sensitive, potentiometric photoluminescent dyes *etc.*, for varied uses. Besides, a photoluminescent measurement can be undertaken in various modalities such as lifetime and photoluminescent resonant-energy-transfer (PRET) measurement [72,73,74]. This versatile technique is adaptable to various situations.

Through the wide choices of photoluminescent probes and simultaneous optical excitation, photoluminescent detection is an ideal technique for multiplexed and high-throughput immunosensing, such as antibody microarrays. The photoluminescent intensity can be enhanced with metal nanostructures [39] and photonic crystals [40] fabricated on the sensor. Li *et al.* [41] reported a nanostructured aluminium-oxide (NAO)-based photoluminescence platform that enhanced a photoluminescent signal up to 100 fold and promoted the photoluminescent-based immunosensing with high throughput. Protein A and fluorophore-labeled immunoglobulin G (IgG) were used to demonstrate that the IgG of programmable concentrations was confirmed with the photoluminescent images on the microfluidic device. The sensitivity of the device as a concentration to detect IgG can be as small as 20 pg/mm^2^ with a photoluminescent microscope. This photoluminescence-enhancing technology provides a powerful tool for photoluminescent-based immunosensing.

## 3. Label-Free Immunosensing

### 3.1. Change of Refractive Index

The optical detection methods mentioned above require either enzymatic or photoluminescent probes labeled on the detection antibody. Not only does the labeling impose additional cost and time, but also the structural modification might interfere with the binding of an antibody to an analyte. A label-free immunosensor is undoubtedly more economical and allows a detection in an unperturbed state. The label-free optical immunosensing typically exploits the local changes of refractive index (RI) induced with an antibody-antigen binding event [75,76]. The refractive indices of an aqueous solution and a protein are 1.33 and 1.5, respectively; the binding of an antibody on the sensor surface causes the local refractive index to shift from 1.33 to an effective refractive index in a range from 1.33 to 1.5 depending on the density of the antibodies bound on the surface. According to the Fresnel equation [77], the phase and amplitude of a reflected and transmitted beam at the interface depend on the refractive index and the thickness of each underlying layer. RI changes thus affect various optical phenomena such as reflection and interference at the interface, which can then serve as approaches to probe RI changes. Examples of these are ellipsometric [78] and interferometer-based immunosensors [79,80]. Another case is the existence of a particular resonant mode, which condition is sensitive to the interfacial RI changes. A surface-plasmon-resonance (SPR) immunosensor is a representative example and is examined in detail below. Other examples of resonance-based sensing include a ring resonator, resonant mirrors and metal-clad waveguide. Among all label-free immunosensors, an SPR-based biosensor has been commercialized and gained popularity in the biomedical research community. Although a label-free approach has labor-saving benefits, an adsorption of molecules other than the target antibodies cannot be discerned; the responses associated with non-binding events are the major sources of error for immunosensing of this type. For this reason, sample preprocessing and experimental controls are crucial steps in label-free immunosensing.

### 3.2. Surface-Plasmon-Resonance (SPR) Immunosensor

**Figure 3:**
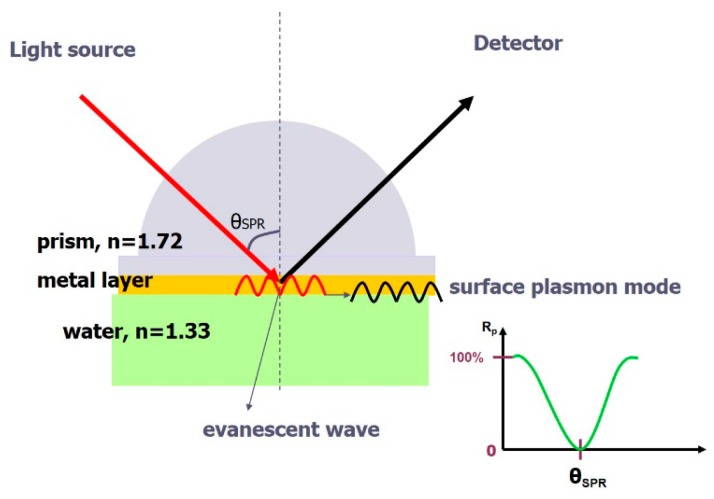
A prism-based SPR sensor with a gold film as sensing layer. The light is absorbed by surface plasmons at a particular resonant angle.

A surface plasmon (SP) is an electromagnetic resonant mode existing at a metal-dielectric interface [81,82,83]; this surface mode is closely related to the refractive indices of the two adjacent media. It carries a mathematic form similar to that of the evanescent wave; the generated electric field decays exponentially away from the interface (Figure 3). As a result, the sensing of a surface-plasmon resonance (SPR) is localized at the interface, excluding other reactions occurring in the bulk region. The concomitant evanescent wave with total reflection is typically employed to excite SP; this excitation requires the match of a wave vector (*k*) at a designated wavelength (λ). The wave vector of the evanescent wave can be modulated on adjusting the incident angle of the beam; when *k* matches that of the SP, a resonance occurs and light is absorbed and transformed to an SP. A standard SPR curve appears in Figure 3. The SPR sensor uses glass as a substrate and a thin layer of gold, typically 50 nm, is coated thereon. The SP lies at the gold-medium interface, and thus the sensing surface; upon the binding of antibodies, the waveform of SP is perturbed and the resonance curves then shift in response to the changes in SP. The reflectance change taken at a fixed angle can be an indicator of binding, proportional to the amount of analyte. Besides the planar SPR sensor, metallic nanoparticles exhibit SPR effects and can serve as immunosensors. The SP present at a metal-ambient interface absorbs light at a specific wavelength; the resonance frequency depends on the shape of the nanoparticle and the ambient refractive index [84]. The general design immobilizes an antibody on a gold nanoparticle; the binding of antibodies and an antigen initiates the aggregation of nanoparticles, which exhibit a surface-plasmon resonance at another frequency [85].

A planar SPR immunosensor can be expanded to an imaging mode (SPRI), which takes the reflection intensity as an indicator of binding and binding quantities. This simple setup has a capability of carrying highly sensitive immunosensing and high-throughput screening. It saves the labor of labeling an antibody with a photoluminescent probe; the antibodies can be regenerated for multiple use. A kinetic binding curve can be acquired in real time on an SPR immunosensor, which can be used to investigate the specific binding between an antibody and an antigen. Although SPRI is capable of incorporating multiple antibodies in parallel for detection, current commercial instruments are limited to a single analyte stream on a multiplexed sensor surface. Integration with microfluidic devices fully realizes the potential of SPRI in multiplexed and multi-analyte screening. Ouellet *et al.* [44] developed an integrated microfluidic array consisting of 264 addressable chambers with each isolated by microvalves and then connected by a serial network for simultaneous measurement of up to six analytes. Besides planar SPR sensors, colorimetric change from metallic nanoparticles is an exhibition of SPR effects; this mechanism enables the design of a particle-based SPR sensor. Gold nanoparticles have long been used in a lateral flow device for rapid and disposable immunosensing [86]; the most popular example is a pregnancy test strip. Recent microfluidic designs further implement gold-nanoparticle sensing on newer materials such as cheap polyester cards [87] and chromatographic papers [88].

## 4. Microfluidics

Microfluidics offers an automatic way to dispense, to merge and to mix small volumes of samples for immunosensing [89,90]. The future development of immunosensing in a medical laboratory aims at rapid, sensitive and multiplex tests with a lancet blood sample. This condition requires sensitive optical detection with appropriate microfluidics integrated on the sensor. One clear goal of microfluidic immunosensing is to perform POC screening with the least support of other equipment. Power-free microfluidics is a promising tool for this purpose and has gained much attention from both academia and industry. Interesting designs of immunosensors using power-free and passive microfluidics, such as paper-based microfluidics and lateral flow devices, have been created, and are introduced in Section 4.1, following. For more accurate manipulation of a liquid sample, as required for a sensitive analysis in a medical laboratory, active and well controlled microfluidics are preferable. Popular designs in this category include Polydimethylsiloxane (PDMS) microchannels with micropneumatic or electrical pumping, compact disk driven by centrifugal force, and digital microfluidic techniques such as electrowetting-on-dielectric (EWOD). The EWOD technique especially performs well in multiple liquid processing, which includes dividing, separating, and transporting a tiny droplet for multiple detection with a simple setup, easily integrated with other optical components. Recent reports of the use of EWOD to manipulate an analyte in optical immunosensing are reviewed in the last Section 4.2.

### 4.1. Paper-Based Microfluidics (μPADs)

Paper, composed mainly of cellulose fiber, wicks an aqueous liquid and transports a liquid passively. It has long been used in chromatography to separate and to identify mixtures of small molecules, amino acids, proteins and antibodies [91]. Paper can be chemically modified with various functional groups that can covalently bind to proteins or DNA, or small molecules. Hydrophobic materials such as wax can be infused into sheets of paper as a barrier to create hydrophilic channels of desired patterns. The pattern and thickness define the dimension of the microfluidic channels on paper. The capillary property of cellulose fibers allows fluids to wick along the channels; there are some factors affecting the rate of wicking, which include the dimensions of the channel, the characteristics of the paper and the ambient temperature and humidity. Besides delivering flow through patterned microfluidic channels, the cellulose matrix of paper can be a solid support for liquid processing, such as filtering samples or performing chromatographic separations. Paper-based microchannels have several advantages over conventional PDMS microchannels as they are cheaper and easier to be fabricated. Paper-based devices are also biodegradable and can be mass-produced to meet the large-scale demand of POC screening.

**Figure 4:**
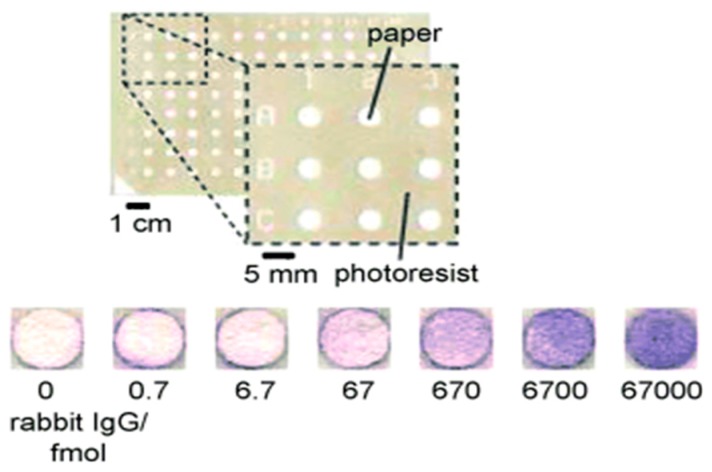
A 96-microzone paper-based ELISA [92]. Reproduction of the figures is made with permission of Wiley.

Microfluidic paper-based analytical devices (μPADs) constitute POC diagnostic devices of a new class specifically designed for use in developing countries. A first paper-based ELISA was devised by Cheng *et al.* [92]; 96-well microzones, resembling a 96-well ELISA plate, were fabricated on paper using photolithographic processing; the hydrophobic photoresist layer confined the liquid reaction to the microzone. The main advantage of paper ELISA over a conventional plastic well is the saving of cost and time. In conventional methods, larger volumes of analytes and reagents (20–200 μL) are required; incubation and blocking duration for each step is long (≥1 h per step) because the analyte requires time to diffuse to the surface of the well. In comparison with the plastic well, paper offers a capillary support to deliver the analyte, thus decreasing the reaction duration. Each microzone requires only 3 µL reagent; the test is completed within an hour, as opposed to 2 h in conventional methods. The colorimetric result is measurable with a desktop scanner (cost $100), rather than an expensive microtitre plate reader. One disadvantage of paper-based ELISA is the decreased sensitivity, which is 54 fmol/zone, one tenth of that obtained with an ELISA plate. This effect is possibly due to the decreased period of antibody-antigen incubation (lower loading) or non-specific interactions between antibodies and cellulose fibers (Figure 4).

**Figure 5:**
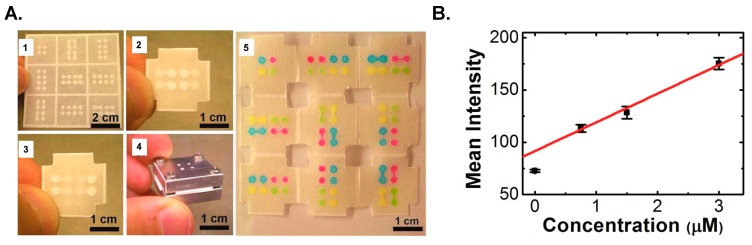
Paper-based microfluidics [94]. (**A**) A 3D paper-based immunosensor using an origami method: **1**. Photolithographically patterned channels on chromatography paper; **2**. Top layer of the device; **3**. Bottom layer of the device; **4**. Aluminium housing; **5**. Colored solutions were injected into designated channels. (**B**) Photoluminescent detection on the paper surface. Reproduction of the figures is made with permission of American Chemical Society.

Paper-based ELISA expands the popular lateral-flow immunosensing to a two-dimensional multiplexed microfluidic immunosensing system. 3D paper-based devices have even more powerful capability, in which μL volumes of samples can be injected from a single inlet point into arrays of detection zones numbering up to thousands [93]. The fluids are distributed vertically and laterally; one stream travels across another without mixing. The 3D paper based microfluidic channels are created by stacking alternating layers of paper and water-impermeable double-sided adhesive tape. The stacking is patterned to allow the liquid flow within the layers of paper, but this design allows only colorimetric detection on the surface layers (top and bottom). Liu *et al.* [94] devised a 3D paper microfluidic device assembled with origami methods (see Figure 5). A piece of chromatography paper can be patterned with channels, reservoirs and frames in a single lithographic step; the paper is then folded along the frames in a sequence, which ensures that channels and reservoirs are aligned to build the 3D connection. Solutions are injected into four holes on the top. The paper is eventually unfolded so that all reservoirs are accessible for colorimetric analysis. Not only did Liu *et al.* propose a novel origami 3D design but also it is the first 3D paper-based immunosensor to adopt photoluminescent sensing, which detection typically offers greater sensitivity and smaller detection limits than simple colorimetric measurements. The photoluminescent detection is based on a dye, epicocconone, which exhibits enhanced photoluminescence in the presence of the analyte protein BSA (bovine serum albumin). Only 1.0 μL of a buffered epicocconone solution is required for the spotting on a detection reservoir; then the analyte BSA is injected from the inlet and reaches the reservoir to react. The layer is then scanned with a photoluminescent imager with resolution 100 μm within 1 min.

The primary detection methods for a paper-based immunosensor are still the qualitative colorimetric methods based on visual comparison with the naked eye or camera telephone. Quantitative analysis is important when the level of an analyte is clinically significant. Other methods such as chemiluminescence provide more precise and sensitive detection and concurrently retain simplicity, portability and modest cost of a paper device. Ge *et al.* [95] developed a paper-based ECL immunosensor on screen-printing carbon electrodes directly on paper; reservoir patterns were first created with wax printing. Carbon working electrodes were then screen-printed on a piece of square paper; another paper was printed with Ag/AgCl reference and carbon counter electrodes. The two papers were then aligned and stacked to form the reservoirs of the electrochemical cells, in total eight electrodes. A panel of biomarkers was screened with this design. With the aid of a chemiluminescent detector, eight working electrodes were sequentially triggered to produce ECL and detected. The linear dynamic range is 0.5–100 ng/mL. This device provides fresh opportunities for sensitive and precise paper-based immunosensors.

The possibility of label-free detection on a paper-based device can be envisaged from the work of Tseng *et al.* [96], involving a plasmonic sensor fabricated on paper using photothermal effects. Chemically synthesized gold nanoparticles have long been used as a sensing platform. This paper-based plasmonic gold-nanoparticle sensor was fabricated with an alternative method: a gold film was first deposited on a paper and laser-induced annealing was performed on the surface; the metal film absorbed the photon energy and converted it into thermal energy, which induced a local formation of nanoparticles. Using this nanostructure as a sensing surface with flow facilitated by the paper offers the possibility of a paper-based plasmonic immunosensor.

### 4.2. Electrowetting-on-Dielectric (EWOD) Digital Microfluidic Devices

Digital microfluidics (DMF) is an emerging droplet-manipulation technique that depends on electric forces, such as electrowetting-on-dielectric (EWOD) or dielectrophoresis (DEP) [97,98]. These forces are caused by the potential difference between electrodes from an electrode array, which are coated with a dielectric and a hydrophobic insulator layer [99,100,101,102] on indium tin oxide (InSnO) glass or printed circuit boards (PCB). Electrowetting-on-dielectric (EWOD) digital microfluidic devices have been endorsed in many reports as a powerful platform for biological and biomedical research, including proteomic analysis [103,104,105,106], single-cell analysis [107,108,109,110], immunoassays [111], and clinical diagnostics [112].

On a DMF device, the small droplets can be individually operated to mix varied reagents, to incubate a reaction mixture, to split a droplet, to wash the modified surfaces or magnetic beads for immunosensing and finally to discard to a reservoir. Because each droplet is controlled with EWOD or DEP forces, complicated channels and external elements (such as pumps or valves or mechanical mixers) are unessential [113]. The comparative advantages of DMF over channel-based microfluidic devices include a simple device configuration, ease of modular interfaces for another integrating system, less requisite total sample volume for assay, less reagent consumption and a high potential for automation [109]. Much attention has thus been paid to the biological and biomedical application of DMF. Here we focus on immunosensors using DMF.

The immunosensing depends on a specific antibody-antigen interaction. In this process, multiple reagents with an antibody, a secondary antibody (conjugated with a tracer, such as HRP, photoluminescent dye or microparticles) and substrates (colorimetric or optical signal) are incubated, respectively; thorough washing with a washing buffer is essential. The DMF device has the advantages stated above to integrate the complicated processes on a device. The multiple droplets with varied reagents for immunosensing might be manipulated with EWOD or DEP forces through designed electrode arrays on the DMF device. The electrodes are coated with a hydrophobic insulator layer, such as Teflon-AF (amorphous fluoroplastics), for smooth operation of a droplet. Since direct immobilization of an antibody or antigen on a Teflon surface is difficult, immunosensing on a DMF device requires an additional solid-phase immobilization, such as magnetic beads [43,114] or surface modification to remove the surface hydrophobicity [115]. Sista *et al.* [43,114] used magnetic beads modified with antibodies to detect human insulin, interleukin (IL)-6 and troponin I from whole-blood samples on a DMF device. Vergauwe *et al.* [116] used magnetic beads to detect the presence of human IgE; the sensitivity of the device to detect IgE is at concentrations of about 150 nM. In another example, the device surface was directly modified; the antibodies were captured onto the hydrophobic surface of a DMF device. Miller *et al.* [115] introduced a surface-based immunoassay using DMF to detect the model analyte human IgG. Ng *et al.* [117] introduced novel magnetic particle-based immunosensing on a DMF device to detect thyroid stimulating hormone (TSH) and 17β-estradiol (E2). These powerful techniques have a great potential for the quantitative analysis of biomarks for diagnosis. The enzymatic chemiluminescent reaction was typically integrated on the DMF device [43,114,117] for optical detection. The photoluminescently labeled probes [115,116] and electrochemical detection [118] were also used. A label-free SPR technique was integrated on DMF devices [45,46]; Malic *et al.* revealed a dynamically configurable microarray SPR sensing on a DMF platform for real-time DNA hybridization. The application of SPR on a DMF platform might achieve a high-throughput screening process.

**Figure 6:**
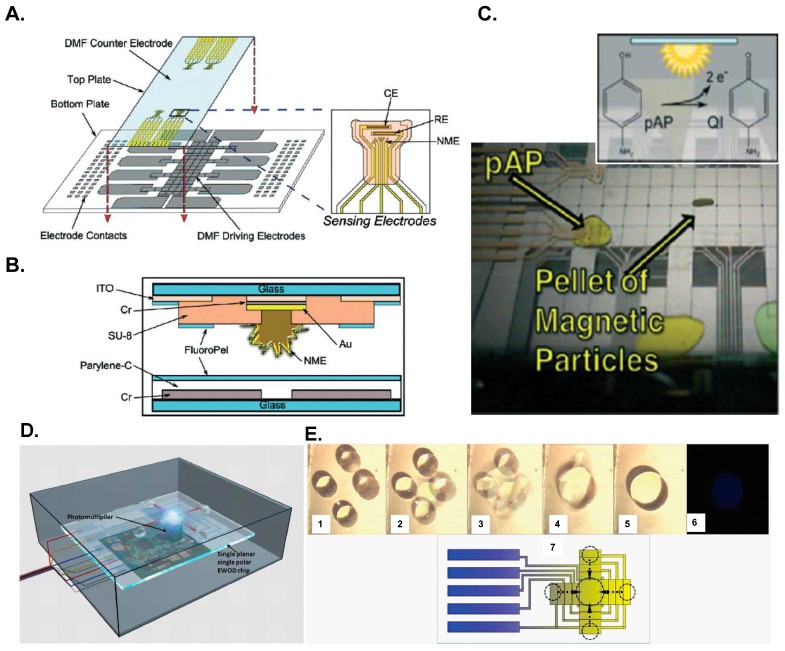
DMF further integrated another platform to perform immunoassays as described in: (**A**–**C**) Rackus *et al.* [36]; (**D**,**E**) Zeng *et al.* [62]. (**A**) DMF device with integrated nanostructured microelectrodes; (**B**) Schematic of a cross section of a DMF device; (**C**) Electrochemical measurements of the DMF device; (**D**) Schematic of the DMF chemiluminescent detector; (**E**) Mixing process on the DMF device (**1**–**5**); chemiluminescent photo (**6**) and schematic diagram (**7**). Reproduction of the figures is made with permission of Royal Society of Chemistry.

DMF has further integrated other techniques to perform immunoassays for sundry biomarkers. These DMF devices have great advantages to implement rapid and inexpensive instruments for diagnosis. Shamsi *et al.* [119] introduced a novel DMF device for magnetic microparticle-based immunosensing with a simple colorimetric detection to detect thyroid stimulating hormone (TSH); the sensitivity of this platform is 2.4 μIU/mL for the tested clinical applications. Rackus *et al.* [36] reported the first integration of nanostructured microelectrodes (NME) with a DMF platform to execute an electrochemical immunosensing for rubella virus (see Figure 6A); the sensitivity of the detection of this immunosensing is 0.07 IU/mL (5.7 μg/mL). Tsaloglou *et al.* [120] described a heterogeneous immunoassay with magnetic beads on a DMF platform to detect the cardiac marker Troponin I (cTnI); the sensitivity of the detection of this immunosensing is 2.0 ng/mL. Zeng *et al.* [62] reported the first experiment to integrate a portable prototype of a CL detector on a DMF platform (see Figure 6B); they manipulated the ball-like droplet to focus better the enzymatic chemiluminescence and thus enhanced the detection sensitivity of the optical signal. The detection limit was 0.01 mM H_2_O_2_. These integrated platforms shed light on highly sensitive CL detection for portable diagnostic devices [36,121,122].

Paper-based devices are mass-producible and disposable; they are suitable for rapid, single use and point-of-care screening. As opposed to paper-based microfluidic immunosensors, the EWOD-based device has more precise control of flow volume and can easily draw multiple droplets from the reservoir for multiplex sensing without elaborative reconstruction of sensor and system. DMF devices employing EWOD have more potential to be developed as desktop immunosensors with high sensitivity and very low sample volume. Compared to other microfluidic platforms, immunosensing using EWOD has not been fully explored yet and the currently reported LOD levels of EOWD-based immunosensors are not satisfactory (μg/mL level, Table 1). However, future development will incorporate other highly sensitive optical detection, such as ECL, in the EWOD- based system. As EWOD-based system has the advantages of simpler device configuration, easy integrating system and high potential for automation, it holds promise for future highly sensitive microfluidic immunosensors.

## 5. Conclusions

This review presents an overview of recent advances in opto-microfluidic immunosensing. Optical detection has long been the most popular detection technique for immunosensing. The advantages of the microfluidic immunosensor devices include decreased requirement of reagents and samples, small power consumption, small size, compact system, modest cost and a high potential for automation. The optical immunosensing process might be miniaturized and integrated with microfluidics to make compact lab-on-a-chip immunosensors. We believe that these integrated platforms of various opto-microfluidic immunosensor designs will fulfill future expectations for portable point-of-care immunosensing diagnostics.
